# Social Media and out of Home Food Selection and Health Promoting Choices: A Generational Perspective in Poland

**DOI:** 10.3390/nu18162727

**Published:** 2026-08-20

**Authors:** Andrzej Soroka, Agnieszka Godlewska, Anna Katarzyna Mazurek-Kusiak

**Affiliations:** 1 Faculty of Medical and Health Sciences, University of Siedlce, Boleslawa. Prusa 14, 08-110 Siedlce, Poland; andrzej.soroka@uws.edu.pl (A.S.); agnieszka.godlewska@uws.edu.pl (A.G.); 2 Department of Tourism and Recreation, University of Life Sciences in Lublin, Akademicka 15, 20-950 Lublin, Poland

**Keywords:** social media, food choices, gastronomic market, intergenerational differences, consumer behaviour, discriminant function analysis

## Abstract

Objective: This study investigates the relationship between social media use and consumer purchase intentions or out-of-home food choices in the Polish gastronomic market, focusing specifically on variations across generations. Methodology: Data were collected between May and July 2024 through a diagnostic survey using the Computer-Assisted Web Interviewing (CAWI) technique (N = 1099). Respondents were recruited via a non-probability quota sampling approach based on strict demographic inclusion criteria. To test the research hypotheses regarding generational variations in market behaviour, a multivariate discriminant function analysis was performed using Statistica 13.1 PL. A preliminary pilot study (N = 30) confirmed the initial questionnaire readability and overall consistency (Cronbach’s alpha = 0.87), while subscales were treated independently during the main analysis. Results: Multivariate models were found to be highly significant, revealing clear differences between age groups. The youngest cohort (aged 18–35) relies heavily on Instagram and TikTok, showing distinct patterns regarding visual triggers such as “instagrammable” aesthetics, digital validation, and menu uniqueness alongside weight loss claims. Conversely, seniors (aged 61 and older) lean unexpectedly towards X. This older segment displays pragmatic, utility-driven motives, searching for detailed textual data about ingredients and the health-promoting properties of food. General product quality and calorie control were identified as universal factors that do not vary by generation. Conclusions and Managerial Implications: The digital transformation of the Polish restaurant industry does not follow a single path. Social media is closely linked to modern customer journeys, with physical dining spots frequently serving as spaces for socialisation. Consequently, restaurant operators should move away from mass communication and adopt a selective omnichannel strategy, where message formats shift from visual appeal to factual, nutrition-oriented text that is tailored to the digital literacy and dietary needs of each generation.

## 1. Introduction and Literature Review

The contemporary catering services market in Poland is undergoing dynamic structural changes. These adjustments stem not only from macroeconomic pressures but primarily from the deep integration of digital tools into consumer eating habits [1]. In today’s networked society, social media platforms have largely replaced traditional channels for gathering nutritional information, evaluating food alternatives, and choosing places to eat out [2]. Social networks no longer serve merely as channels for casual interpersonal communication. Instead, they have evolved into powerful environments for sensory and informational marketing that actively alter how individuals select their food [3,4]. This evolution matches a broader shift towards technology-reliant lifestyles, rewriting how individuals process dietary details and execute out-of-home food choices [5,6,7,8].

Strong competition and growing customer expectations regarding food authenticity, nutritional value and health benefits define the contemporary Polish culinary market [9,10]. Such conditions turn the sector into an ideal landscape for evaluating how digital networks modify public health choices and daily eating patterns [11]. The academic literature highlights that because food is highly experiential and sensory, dining decisions remain uniquely vulnerable to online cues [12]. The consumer journey of the modern restaurant guest, especially within younger demographics, hinges on continuous exposure to mixed visual and textual media, a process that challenges standard nutritional choice models [2,13]. These behavioural changes are consistent with the strong growth of digital commerce and e-health solutions in Poland. In this environment, social media provides a space where consumers connect with culinary brands, analyse their nutritional profiles, and adapt their eating habits across age groups [1].

The way social platforms alter consumer food choices is multidimensional. For instance, these networks run on mechanisms such as online social proof and electronic word of mouth, which effectively lower consumer anxieties regarding food quality and kitchen safety [2]. This digital transformation is shifting the focus from basic, functional nutrition to experiential eating, leaving online culinary experiments, niche dietary models, and aesthetic presentation in the spotlight [14]. The visual styling of meals, often labelled as “instagrammable” food design, helps dictate the market draw of modern eateries and directly shapes immediate sensory expectations [14]. Furthermore, platform algorithms tailor content feeds to individual user profiles, which shortens traditional buying paths and rapidly converts casual screen browsing into active food consumption [6,10].

Social media channels offer direct, public collections of information about food composition, calorie values, and specific health benefits [14,15,16]. This information access plays a major role for the expanding group of health-conscious buyers, shifting their underlying dietary motives and selection rules [11,17,18]. Beyond individual choices, digital phenomena are fostering social interactions based on eating habits. People are seeking inspiration on social media, modifying their diets, and organising group meals tailored to specific culinary trends [2]. Peer feedback and the promotional efforts of online food influencers thus gain extra leverage, actively establishing new sustainable and functional eating habits among Polish consumers [17,19].

Despite an expanding body of literature on digital marketing, a complete understanding of the specific mechanisms that dictate how different social platforms affect food decisions is still lacking.

This study is grounded in the Uses and Gratifications Theory (UGT), which explains how consumers actively choose specific social media channels to meet their information and entertainment needs. In the foodservice sector, researchers often use UGT to show how online reviews and food photos satisfy user needs and shape consumer decisions [20]. This paper extends UGT by analysing how these digital needs and behaviours differ across multiple generations when choosing a restaurant.

Past studies have mostly looked at isolated communication feeds, broad buying intentions, or highly specific demographic groups, mainly teenagers and young adults [1,21]. For instance, recent empirical insights into the Polish marketplace have heavily prioritised the food selection parameters and sustainability views exclusively within Generation Z [21], or evaluated generalised food grocery purchasing patterns via social networks across broad gender variables [6,22].

Researchers have rarely attempted to simultaneously map out how social media exposure alters restaurant selection, desired sensory experiences, raw ingredient awareness, and the core nutritional metrics that guide purchases across multiple age cohorts [1,16,23].

Generational variations in how consumers deploy social platforms within the Polish out-of-home dining sector remain largely overlooked, even with the ongoing shift towards digital consumption. Shifting age dynamics and unequal levels of digital or nutritional literacy across generations lead to wildly different reactions to online food marketing [24]. Breaking down these generational divides is an essential step for building practical healthy eating initiatives that resonate with the values and dietary goals of various age cohorts [1]. Because distinct food habits, peer dynamics, and technology adoption rates split cohorts from Generation Z to older generations, isolating these age-specific details is crucial [15]. This clear gap creates an urgent need for empirical work utilising solid, representatively large sample sizes alongside multivariate analytical frameworks to map consumer trends accurately.

Based on the literature and UGT, we developed four hypotheses.

First, social media gives consumers reviews and social proof, which helps them trust a restaurant. Therefore, we assume:

**H1.**  *Social media exposure has a positive effect on restaurant selection among Polish consumers.*

Second, different age groups have different levels of digital skills and technology habits. This creates a generational divide online. Thus, we hypothesise:

**H2.**  *The impact of social media on restaurant choices differs significantly across age groups.*

Third, dining choices are highly sensory, meaning that food photos on platforms like Instagram create instant expectations about taste. Consequently, we propose:

**H3.**  *Visual content (such as “instagrammable” food design) shapes immediate sensory expectations.*

Finally, health-conscious buyers actively use social media to check ingredients and health benefits before eating out. Based on this, we assume:

**H4.**  *Online information about calories and food benefits influences the choices of health-conscious buyers.*

## 2. Materials and Methods

### 2.1. Research Design and Survey Tool

This study used a cross-sectional diagnostic survey design to examine factors influencing food choices at home. A quantitative survey framework provides a well-established approach to collecting standardised data, building on established traditions in nutrition and public health research [25]. The primary research tool was an original, structured questionnaire that originally contained 14 items. To ensure tight thematic alignment with the core dietary and food choice objectives of this project, we extracted four specific multi-attribute closed-ended questions for the final empirical analysis.

We measured consumer attitudes and the relative weight of dietary choice factors using a standard five-point Likert scale, ranging from one “minimal significance” to five “maximum significance”. This scaling choice is standard in nutritional psychology and consumer research, allowing investigators to convert qualitative consumer preferences into parametric datasets ready for multivariate statistical testing [26].

Developing and testing this metric scale involved a multi-step validation process. First, to clean the text of interpretative ambiguity, improve clarity, and prevent structural errors, a panel of independent academic experts evaluated the initial item pool using the expert judgement technique. Following their feedback, we ran a small pilot study among a preliminary group of 30 respondents. These pilot responses were used to assess the internal consistency of the questionnaire. The overall Cronbach’s alpha was 0.87. Since this value exceeds the standard 0.70 threshold, the tool was considered reliable for the subsequent main data collection, where subscales were analysed independently [27].

To protect data quality and reduce problems like cognitive fatigue or completion rush, the survey software did not impose time limits on respondents. Before opening the form, participants read a briefing on the non-commercial, purely academic nature of the project. We maintained administrative transparency by providing direct institutional email channels to the principal researchers. Participation remained entirely voluntary, and we guaranteed full anonymity to every respondent. The entire research setup followed the ethical mandates for human research established in the Declaration of Helsinki and fully complied with applicable institutional data protection laws.

### 2.2. Data Collection and Sample Segmentation

The empirical fieldwork was conducted over a three-month period between May and July 2024 across all administrative regions in Poland. Participants were recruited using a non-probability convenience quota sampling approach [25] to ensure that the final sample reflected the demographic profile of the adult population in Poland.

To achieve this, quota sampling based on the official Central Statistical Office (GUS) data for Poland was applied. The sample was controlled for key demographic characteristics, specifically gender, age groups, and region of residence. This approach ensured that the structural proportions of the survey participants aligned with the wider adult population of Poland, specifically regarding biological sex and generational cohorts. To ensure data quality, strict inclusion criteria were applied. Eligible participants had to be at least 18 years old, permanent residents of Poland, regular consumers of out-of-home gastronomic services, and willing to provide informed consent electronically before accessing the questionnaire.

Primary data were collected via the Computer-Assisted Web Interviewing (CAWI) method among Polish consumers. The first version of the questionnaire items was developed based on the existing literature on social media food marketing and consumer behaviour. Specifically, questions regarding social media usage and information search were adapted from scales used by Godlewska et al. (2025) [6]. Items were then modified to fit the out-of-home dining sector and translated into Polish. Additionally, the questionnaire included a section named “Key Information About Products”. This section was designed to measure which specific food characteristics interest consumers when browsing social media for dining choices. It focuses on how users evaluate raw ingredients, allergen data, calorie counts, and specific health benefits before selecting a restaurant. These items were selected to link social media marketing directly with the nutritional awareness of different age cohorts. Before the main study, a pilot test was conducted with 15 respondents to ensure clarity and validity. The survey was distributed through two main channels: email invitations sent via university networks and links shared on major social media platforms (Facebook, X, and Instagram). Following the fieldwork phase, the dataset was cleaned to remove incomplete responses, straight-lining patterns, and logical inconsistencies. This process resulted in a final sample of *N* = 1099 valid adult responses for statistical analysis.

The sociodemographic characteristics of the final sample (*N* = 1099) are detailed below:

Biological Sex: The sample showed a balanced gender distribution, including 596 female respondents and 503 male respondents.

Generational Cohorts: Respondents were divided into three age groups representing distinct socioeconomic and digital backgrounds:

Young Adults: A cohort linked in the nutritional literature to developing dietary autonomy, financial independence, and specific online behavioural patterns regarding plant-based or functional food choices in Poland [1].

Established Adults: The largest demographic segment, characterised by stable career and purchasing structures, who combine traditional nutritional values with modern digital consumption channels [28].

Seniors: A segment representing individuals transitioning into retirement, whose dietary choices focus heavily on health preservation, and who use digital spaces primarily for information and communication [29].

Educational Attainment: The sample skewed towards higher qualifications, with 79.45% of respondents holding a university degree (Bachelor’s, Master’s, or equivalent). Secondary education was reported by 16.10% of the sample, while the remaining 3.45% had vocational or primary education (Table 1). The final sample has a high proportion of university-educated respondents (79.45%). This overrepresentation reflects self-selection and the digital literacy biases common in online survey channels and university network distribution. In this study, this profile is interpreted within the context of health-conscious food trends and digital restaurant marketing in Poland, which are heavily driven by highly educated, urban demographics. Regarding platform usage, senior cohorts (aged 61+) engaged with X primarily for informative and text-based gratifications rather than entertainment, aligning with the Uses and Gratifications Theory (UGT). This specific digital behaviour explains their participation through diversified social media feeds.

### 2.3. Statistical Analysis

Quantitative data analysis was performed using Statistica 13.1 PL (StatSoft Inc., Tulsa, OK, USA). To test the research hypotheses (H1 to H4) regarding intergenerational variations in out-of-home nutritional choices, a multivariate discriminant function analysis (DFA) was applied. Discriminant function analysis (DFA) was chosen because it helps identify which variables best differentiate distinct groups. In this study, we used DFA to find out which social media habits and food choices separate our three age groups. This method allows us to directly test the generational differences stated in Hypothesis 2.

This approach helped identify which specific attributes, namely sought nutritional information, sensory experiences, or restaurant selection drivers, contributed most to differentiating the three consumer age cohorts [30].

Classification function equations were calculated independently for each age group. Before running the analysis, key assumptions were verified, including univariate normality within each subgroup and the homogeneity of variance-covariance matrices. Although the sample sizes were asymmetrical across generations, discriminant function analysis is highly robust against deviations from multivariate normality in large samples (*N* > 1000), ensuring unbiased estimations of discriminatory power [31].

The statistical significance of the overall discriminant model and the unique contribution of individual behavioural and informational variables was evaluated using Wilks’ Lambda. For all statistical tests, the significance threshold was set a priori at *p* < 0.05 [18].

## 3. Results

### 3.1. Verification of Hypothesis H1

To verify hypothesis (H1), which assumes significant intergenerational differences in using social media for restaurant selection and food perception, a discriminant function analysis was conducted. The three age cohorts served as the grouping variable, while individual social media platforms were treated as independent variables. The overall model was highly significant (Wilks’ Lambda = 0.451, F = 19.048, *p* < 0.001), demonstrating that the selected variables possess a strong capacity to differentiate the studied consumer groups. The classification accuracy for the original sample was 81.4%**,** demonstrating high predictive validity, and remained stable at 79.8% during the cross-validation procedure. Box’s M test confirmed the homogeneity of covariance matrices (*p* > 0.05).

An assessment of the partial Wilks’ Lambda values and F tests established the hierarchy of variables based on their discriminatory power. Instagram emerged as the most powerful discriminator among the age cohorts (F = 219.058, *p* < 0.001), followed by X (F = 54.346, *p* < 0.001) and YouTube (F = 17.561, *p* < 0.001). Facebook exhibited the lowest, yet still statistically significant, discriminatory power within the model (F = 3.701, *p* = 0.025) (Table 2).

Analysis of the classification function coefficients revealed distinct social media profiles across the age groups. For X, the highest coefficient was recorded among respondents aged 61 and older (3.529), indicating that this platform strongly characterises the senior cohort. This counter-intuitive pattern stems from the specific demographic profile of this older sample, which is highly educated. These senior respondents utilise X primarily as a source for text-dense, fact-based information, short articles, and real-time news regarding food safety and ingredients, rather than casual entertainment. Therefore, this finding represents a genuine demographic nuance within this highly educated subgroup rather than a statistical artefact. Regarding Facebook, the highest classification value was found in the 36–60 age bracket (1.215), followed by the youngest group. A similar trend emerged for YouTube, where the classification coefficient for the 36–60 age group reached 0.827, showing a descriptive advantage over the other two brackets. Conversely, the youngest consumers (aged 18–35) exhibited a different behavioural pattern, dominated by highly visual platforms. The classification coefficients confirmed that the use of TikTok (0.532) and Instagram (1.262) is a distinctive feature of the youngest segment, with these parameters dropping sharply in the older cohorts. Consequently, these statistical distributions fully support the positive validation of hypothesis (H1) (Table 2).

### 3.2. Verification of Hypothesis H2

The next stage of the empirical analysis verified hypothesis (H2), which examines intergenerational differences in the perceived importance of factors influencing restaurant selection under the influence of social media content. A second multivariate discriminant function analysis model was applied for this purpose. The overall model was highly significant (Wilks’ Lambda = 0.411, *F* = 19.048, *p* < 0.001), confirming that the selected explanatory variables are well suited for group differentiation. The model achieved an overall classification accuracy of 83.2% for the original dataset and 82.1% in the cross-validation analysis, indicating strong model stability. The assumption of equal covariance matrices was met (Box’s M test, *p* > 0.05).

Based on the partial Wilks’ Lambda values and F tests, the factors with the strongest discriminatory power among the age cohorts were the number of likes on a post presenting a dish (F = 54.801, *p* < 0.001), the location of the establishment (F = 48.652, *p* < 0.001), and price (F = 26.657, *p* < 0.001). The only variable that failed to exhibit statistically significant discriminatory power was the type of cuisine offered (F = 1.303, *p* = 0.273), suggesting this criterion holds universal importance regardless of respondent age (Table 3).

Analysis of the classification function coefficients revealed distinct decision-making profiles across the generations. The youngest consumer group (aged 18–35) showed the highest sensitivity to financial, visual, and social indicators on social media. Online content most strongly influenced their choices regarding price (2.491), interior design (0.876), and menu uniqueness (0.714). Furthermore, this cohort was heavily influenced by online popularity metrics, recording the highest classification values for establishment popularity (0.605), dish customisation options (0.387), and the number of post likes (0.728). Information regarding the physical location of the restaurant also had the strongest impact on this youngest group (0.687). A distinctly different motivational structure characterised the oldest consumer segment (aged 61 and older). For this cohort, health aspects promoted on social media emerged as the key differentiating feature, reaching the highest coefficient in the model (0.912). Interestingly, menu uniqueness (0.602) and the number of likes (0.417) were also relatively important factors for seniors, which may reflect a desire for verified yet novel culinary experiences. Conversely, the profile of respondents aged 36–60 was the most balanced and the least susceptible to digital influence, yielding moderate coefficient values across most fields. These findings provide a robust basis for the positive validation of hypothesis (H2) (Table 3).

### 3.3. Verification of Hypothesis H3

The next stage of statistical inference verified hypothesis (H3), which examines intergenerational differences in the types of food product information consumers seek on social media. The multivariate discriminant function analysis model, incorporating seven independent variables, was highly significant overall (Wilks’ Lambda = 0.415, F = 14.037, *p* < 0.001), indicating that the selected diagnostic features are well suited for group differentiation. Model diagnostic parameters confirmed the validity of the analysis, as Box’s M test indicated homogeneous covariance matrices (*p* > 0.05). The linear function correctly classified 78.9% of the original cases and sustained a stable classification accuracy of 77.5% under the leave-one-out cross-validation procedure, confirming distinct group centroid distribution across the cohorts.

Verification of the partial Wilks’ Lambda values and F tests established the hierarchy of variables based on their discriminatory power within the Polish gastronomic market. Information regarding the use of regional products emerged as the strongest discriminator (F = 35.715, *p* < 0.001), followed by weight loss attributes (F = 28.893, *p* < 0.001). Significant discriminatory power was also found for variables describing knowledge about elimination diets (F = 18.765, *p* < 0.001), the health benefits of culinary components (F = 8.118, *p* = 0.001), and detailed ingredients (F = 7.722, *p* = 0.001). Two variables did not show significant discriminatory power within the model: the calorie count (F = 1.194, *p* = 0.303) and the quality of ingredients (F = 1.128, *p* = 0.327). This suggests that attention to quality and general calorie control represent universal concerns among Polish consumers, regardless of their age groups (Table 4).

Analysis of the classification function coefficients revealed clear differences in the informational focus of the age groups on social media. For the youngest consumer cohort (aged 18–35), social media platforms serve primarily as a source of information related to body image and ethical or environmental concerns. This group recorded the highest classification coefficients regarding weight loss attributes (1.402), preferences for regional and traditional food (0.454), and menus tailored to elimination diets, including vegan or vegetarian options (0.336). A contrasting informational profile emerged for the senior segment (aged 61 and older). Older online users focus primarily on the nutritional and health-related aspects of their diet. The discriminant model indicated that highlighting the health benefits of raw ingredients on social media is of primary importance for this group (0.712). Furthermore, seniors closely examine the full list of ingredients used in a dish, as shown by their dominant classification coefficient (1.184). Consumers aged 36–60 again exhibited intermediate characteristics, balancing health-promoting aspirations with dietary restrictions. These distinct informational needs across the cohorts fully support the positive validation of hypothesis (H3) (Table 4).

### 3.4. Verification of Hypothesis H4

A contrasting informational profile emerged for the senior segment (aged 61 and older). Older online users focus primarily on the nutritional and health-related aspects of their diet. The discriminant model indicated that highlighting the health benefits of raw ingredients on social media is of primary importance for this group (0.712). Furthermore, seniors closely examine the full list of ingredients used in a dish, as shown by their dominant classification coefficient (1.184). Consumers aged 36–60 again exhibited intermediate characteristics, balancing health-promoting aspirations with dietary restrictions. These distinct informational needs across the cohorts fully support the positive validation of hypothesis (H3). The assumptions for this fourth multivariate model were fully met, with Box’s M test showing non-significant differences in covariance matrices (*p* > 0.05). Group centroid metrics confirmed optimal operational distance between the segments. The original classification rate achieved an accuracy of 82.5%, which demonstrated high mathematical stability, with an 80.9% accuracy score verified via the cross-validation procedure.

Verification of the partial Wilks’ Lambda values and F tests revealed that seeking information about promotions and culinary events was the strongest discriminator among the age cohorts within the Polish gastronomic market (F = 18.919, *p* < 0.001). Significant discriminatory power was also found for variables such as new menu items (F = 11.002, *p* < 0.001), tracking new dietary trends (F = 10.426, *p* < 0.001), and the desire for flavour experimentation (F = 10.255, *p* < 0.001). Seeking cooking inspiration with loved ones made the smallest, yet still statistically significant, contribution to the model (F = 6.187, *p* = 0.002) (Table 5).

Analysis of the classification function coefficients revealed unique behavioural profiles across the age groups. The desire for flavour experimentation emerged as the dominant culinary experience sought on social media across all age cohorts, yielding the highest coefficient values in every segment. However, this factor most strongly characterised the youngest consumers (aged 18–35), for whom the parameter reached its maximum value (1.411). For this youngest cohort, social media platforms serve primarily as a source of market information and a tool for socialisation. This segment recorded the highest classification coefficients in the model for variables describing the online search for promotions and culinary events (0.783), tracking new dietary trends (0.345), and seeking cooking inspiration with loved ones (0.443). Analysis of the coefficients for tracking new menu items revealed a non-linear structure. The highest value was recorded among the youngest respondents (0.782), yet the senior segment (aged 61 and older) emerged as a strongly driven group by this need (0.682). This suggests that older consumers who actively use social media platforms utilise them to verify product innovations before visiting an establishment. Conversely, consumers aged 36–60 exhibited the lowest susceptibility to novelties, promotions, and trends, confirming a higher degree of conservatism and adherence to established gastronomic habits. These differences in the structure of sought culinary experiences fully support the positive validation of hypothesis (H4) (Table 5).

## 4. Discussion

By employing a multivariate discriminant function analysis, this research moves beyond descriptive statistics to identify the behavioural, psychological, and informational factors that govern how different age cohorts utilise social media when selecting out-of-home food options [27,30].

The dominance of visual platforms, especially TikTok and Instagram, among the youngest cohort (ages 18–35) reflects a preference for fast-paced digital content. For age group Z and younger millennials, choosing a restaurant is no longer a purely utilitarian transaction; it is closely linked to building a digital identity and sharing a lifestyle. This behavioural pattern aligns with the “social media nudging” model presented by Charry and Tessitore (2021) [32], who observed that specific digital metrics, such as a large number of followers, act as a powerful social trigger. This digital confirmation acts as a catalyst that can change younger consumers’ perceptions and intentions toward recommended healthy food categories.

Visual networks serve as key spaces where recommended information shapes gourmet orientation, frequent out-of-home consumption, and variety-seeking inclinations among youth [3,33]. Within the Polish marketplace, this visual exposure operates as a tool for sensory and informational marketing, where peer validation shortens the decision-making process and converts passive screen time into immediate out-of-home dining choices [4,6].

Statistically significant reliance on Facebook and YouTube by adults (ages 36–60) indicates a preference for mature, long-form information ecosystems. Rather than seeking quick, aesthetically pleasing content, this group primarily uses digital networks to mitigate risk by verifying peer recommendations, service quality, and practical considerations before dining out.

This matches the multi-channel consumer frameworks outlined by Dwivedi et al. (2021) [5]. Within this segment, digital channels serve as analytical tools rather than hedonic catalysts, prioritising factual parameters such as predictable pricing, ingredient transparency, and accessibility over temporary visual trends. This pragmatic pattern aligns with the socio-demographic predictors identified in Poland by Michalak et al. (2025) [11], where older cohorts place a higher premium on healthfulness, quality, and nutritional safety. Consequently, digital engagement for this demographic is a deliberate process of knowledge validation and critical evaluation to safeguard personal well-being [15,34].

A key finding from the multivariate profiling is the senior segment’s (aged 61 and older) connection with X. This distinct classification weight on text-dense informational environments challenges the traditional academic stereotype of the digitally passive elderly. This shift aligns with the conceptual framework of Anter et al. (2025) [35], whose tracking of “silver” consumers confirmed that older individuals increasingly integrate social media into their daily information practices. Within the Polish socioeconomic landscape, this dynamic mirrors the observations of Zalega (2016) [22] regarding the evolution of silver consumer behaviour, where modern retirees actively move away from passive disengagement towards strategic, quality-of-life-oriented market navigation.

While traditional stereotypes have often confined older users’ social media activity to basic interactions, modern seniors demonstrate more conscious habits. They deliberately leverage specific platform features as tools to explore market innovations and verify product information [34]. In out-of-home dining decisions, this text-driven vigilance functions as a vehicle for nutritional validation and health literacy, allowing older cohorts to screen food environments and safeguard their well-being before making consumption commitments [16].

The structure of the model coefficients further exposes this generational polarisation. The youth cohort (aged 18–35) exhibits extreme sensitivity to visual design indicators, electronic word-of-mouth validation, and post likes. Dining out serves as a visible marker of peer group alignment and social status. Within this demographic, visually structured food arrangements or unique venue interiors operate as a form of social currency, effectively driving immediate out-of-home consumption [6].

In sharp contrast, the senior segment is firmly anchored by the health-promoting attributes of the gastronomic offering. For older consumers, digital networks serve as a protective tool to safeguard physiological well-being and metabolic health, functioning as a mechanism for cross-national nutritional validation [16]. Interestingly, their secondary classification weight on dish uniqueness suggests that modern Polish seniors are moving away from historically conservative eating habits during their leisure time. This dynamic mirrors the observations of Zalega (2016) [22] regarding the evolution of the Polish silver market, where retirees increasingly prioritise quality-of-life parameters, experiential consumption, and active lifestyle expansion over basic commodity acquisition. It also reflects a broader European shift where older populations utilise digital spaces for nutritional validation and healthy ageing screening [16].

The confirmation of hypothesis (H3) reveals a clear dichotomy between the identity-centric nutrition management of youth and the health-focused vigilance of senior consumers. For the 18–35 age bracket, social media search parameters revolve around weight loss attributes and restrictive diets (e.g., veganism or vegetarianism). In this context, digital feeds act as an echo chamber reinforcing peer-group body-image expectations, lifestyle aesthetics, and eco-ethical standards, a phenomenon corroborated in European food choice studies by [2,17]. Within the Polish academic literature, this psychological matrix among younger demographics frequently correlates with anxiety surrounding nutritional composition, driving trends towards selective diet quality management and alternative eating patterns [14].

Seniors, however, apply strict empirical parameters to their online evaluations, prioritising raw component disclosure, health benefits, and ingredient transparency. The informational vigilance observed among the Polish senior cohort (aged 61+), specifically their preference for evaluating detailed ingredients and health-promoting properties, strongly resonates with the findings of Anter et al. (2025) [35]. This confirms that for the silver segment in Poland, social networks have transitioned from casual communication channels into deliberate educational touchpoints used to actively verify food quality and nutritional safety before consumption.

The unexpectedly high association between senior respondents and X (formerly Twitter) is a direct result of recruitment and selection biases. Since the survey was distributed online via Facebook, Instagram, and X, it naturally reached older adults who are already highly digitally literate and active on these platforms. Within this specific, university-educated sample, these senior users deploy X primarily for text-dense, fact-based information rather than entertainment. Therefore, this pattern reflects a unique demographic subgroup of active internet users rather than the general Polish senior population.

A limitation of this study is that it focused primarily on generational cohorts without fully controlling for other potential socioeconomic and behavioural confounders. Factors such as education levels, sex, disposable income, place of residence, eating-out frequency, and overall daily social media usage could also shape dietary choices and digital platform preferences. Future research should implement more complex multivariate models to evaluate the independent and interaction effects of these confounding variables alongside the generational factor.

## 5. Conclusions

The empirical evidence gathered in this study provides a clear insight into how social media shapes the Polish gastronomic services sector. By employing a multivariate discriminant function analysis, hypotheses H1 through H4 were successfully confirmed. The models demonstrate that social media platforms do not impact food consumers uniformly but instead drive a distinct, age-based divide.

The findings reveal that for the youngest cohort (aged 18–35), the digital ecosystem dominated by Instagram and TikTok has thoroughly altered the customer journey. In this group, social platforms fulfil an experiential and social role, shifting the primary motive for dining out from a functional need to social group alignment, status display, and visual appeal. Conversely, the senior segment (aged 61 and older) interacts with digital media through a lens of utility and pragmatism, showing a distinct preference for X. For these older consumers, social networks function as validation tools used to closely inspect ingredients, health benefits, and nutritional values before visiting a restaurant.

Ultimately, while product quality and calorie awareness remain baseline expectations across all age groups, communication preferences, visual triggers, and dining expectations are highly fragmented. Social media spaces have essentially turned modern Polish restaurants into digitalised “third places”. In the contemporary marketplace, entering a physical venue is almost always preceded by a generationally distinct phase of virtual pre-purchase validation.

## 6. Managerial Implications

From a public health perspective, these findings highlight that social media platforms are no longer just marketing tools, but digital environments that shape dietary behaviours. Although this study did not measure actual food intake or metabolic health outcomes, understanding how different generations process nutritional information online is vital for health promotion. For instance, the senior group’s active search for fact-based data on health-promoting properties suggests that digital channels can be effectively utilised for targeted senior nutrition campaigns. Conversely, the youth’s reliance on highly visual platforms and immediate social validation presents both a risk for trend-driven, unbalanced eating habits and an opportunity for public health authorities to deploy visually engaging nutritional interventions where young consumers actively make lifestyle decisions.

The deep generational divergence documented in this research means that restaurateurs and hospitality marketers can no longer rely on broad mass marketing. Instead, they must adopt a targeted, selective omnichannel approach tailored to three distinct behavioural profiles (Table 6).

## 7. Limitations and Future Research

While this study benefits from a large, representatively structured sample (*N* = 1099) and a rigorous multivariate approach, certain boundaries highlight vital paths for future academic study:

Methodological Constraints: The dataset originates from a cross-sectional survey conducted between May and July 2024 via CAWI, presenting a specific socioeconomic snapshot. Future research should adopt longitudinal frameworks to monitor how stable these generational digital behaviours remain across shifting economic cycles or seasonal changes.

Geographical Scope: The empirical field was strictly limited to the Polish gastronomic market. Although Poland serves as a reliable proxy for emerging economies across Central and Eastern Europe, these insights cannot be automatically applied to Western European, North American, or Asian markets where cultural dining habits differ vastly. Cross-cultural comparison studies would significantly enrich the current body of literature.

Technological Shifts: The rapid evolution of platform algorithms, social commerce tools, and the rising role of Artificial Intelligence (AI) in automated food recommendations fell outside the primary scope of this research. Future studies should explore how automated algorithmic profiling and generative AI tools such as conversational search assistants or dynamic, personalised digital menus reshape the buying paths of different demographic cohorts.

## Figures and Tables

**Table 1:** Socio-demographic characteristics of the final research sample (N = 1099).

Characteristic	Category	Number (*N*)	Percentage (%)
**Biological Sex**	Female	596	54.23%
	Male	503	45.77%
**Age groups**	18–35 years	410	37.30%
	36–60 years	556	50.59%
	61 years and older	133	12.11%
**Educational Attainment**	Higher (University degree)	873	79.45%
	Secondary education	177	16.10%
	Vocational or primary education	38	3.45%

**Table 2:** Classification function coefficients for social media preferences.

Type of Social Media	Wilks’ Lambda	*F* Value	*p*	Group: 18–35 Years	Group: 36–60 Years	Group: 61 Years and Older
X (formerly Twitter)	0.453	54.346	<0.001	1.554	2.675	3.529
Facebook	0.432	3.701	0.025	1.052	1.215	1.127
YouTube	0.428	17.561	<0.001	0.453	0.827	0.518
TikTok	0.429	7.658	<0.001	0.532	0.273	0.172
Instagram	0.449	219.058	<0.001	1.262	0.082	0.132
Constant	—	—	—	6.384	5.053	6.957

Note: Statistical significance level of differences at *p* < 0.050. Source: Author’s own analysis based on research material (N = 1099).

**Table 3:** Classification function coefficients for factors determining restaurant selection under social media influence.

Type of Factor	Wilks’ Lambda	*F* Value	*p*	Group: 18–35 Years	Group: 36–60 Years	Group: 61 Years and Older
Prices of dishes in the establishment	0.389	26.657	<0.001	2.491	1.861	1.789
Type of cuisine offered	0.378	1.303	0.273	2.054	1.967	1.845
Interior design of the establishment	0.438	3.376	0.034	0.876	0.618	0.701
Uniqueness of the offered dishes	0.412	7.395	<0.001	0.714	0.383	0.602
Health aspects of the culinary offer	0.412	7.648	<0.001	0.605	0.471	0.912
Popularity of the given restaurant	0.398	5.592	0.003	0.605	0.345	0.234
Customization options for dishes	0.401	5.048	0.006	0.387	0.143	0.153
Number of likes on a post	0.432	54.801	<0.001	0.728	0.093	0.417
Location of the establishment	0.419	48.652	<0.001	0.687	0.023	0.165
Constant	—	—	—	14.839	10.523	11.543

Note: Statistical significance level at *p* < 0.050. Source: Author’s own analysis based on research material (N = 1099).

**Table 4:** Classification function coefficients for key information determining food product purchases.

Key Information About Products	Wilks’ Lambda	F-Value	*p*	Group: 18–35 Years	Group: 36–60 Years	Group: 61 Years and Older
Weight loss attributes of products	0.458	28.893	<0.001	1.402	0.994	0.675
Ingredients used in dish preparation	0.429	7.722	0.001	0.704	0.823	1.184
Number of calories in a dish	0.321	1.194	0.303	0.343	0.473	0.263
Healthy products used in a dish	0.439	8.118	0.001	0.164	0.238	0.712
Regional products utilised	0.476	35.715	<0.001	0.454	0.023	0.264
Elimination diets (e.g., vegan, etc.)	0.432	18.765	<0.001	0.336	0.198	0.036
Quality of utilised products	0.437	1.128	0.327	0.107	0.052	0.087
Constant	—	—	—	5.840	4.567	6.302

Note: Statistical significance level at *p* < 0.050. Source: Author’s own analysis based on research material (N = 1099).

**Table 5:** Classification function coefficients for types of culinary experiences sought on social media.

Type of Culinary Experience	Wilks’ Lambda	F-Value	*p*	Group: 18–35 Years	Group: 36–60 Years	Group: 61 Years and Older
Promotions and culinary events	0.387	18.919	<0.001	0.783	0.461	0.152
New proposals in the restaurant menu	0.376	11.002	<0.001	0.782	0.472	0.682
New dietary trends	0.397	10.426	<0.001	0.345	0.053	0.103
Flavour experimentation	0.323	10.255	<0.001	1.411	1.076	1.067
Cooking with loved ones	0.361	6.187	0.002	0.443	0.187	0.198
Constant	—	—	—	6.329	3.457	4.473

Note: Statistical significance level at *p* < 0.050. Source: Author’s own analysis based on research material (N = 1099).

**Table 6:** Summary of managerial implications for the restaurant sector.

Target Segment/Area	Key Practical Recommendation	Marketing Channel and Content Focus
Young Adults (18–35 years)	Promote modern, niche dietary models and interactive culinary trends.	High-visual platforms (Instagram, TikTok). Focus on aesthetic, “instagrammable” food design.
Established Adults (36–60 years)	Combine traditional nutritional values with clear digital profiles of dishes.	Balanced mix of social proof, Facebook campaigns, and detailed brand websites.
Seniors (61 years and older)	Provide easy access to direct facts regarding kitchen safety and ingredient origin.	Informational posts on Facebook and official blogs with clear, easy-to-read textual summaries.
Health-Conscious Buyers (All groups)	Reduce consumer anxieties by publishing transparent nutritional profiles online.	Public repositories, pinned posts, or social media highlights showing calories and allergen details.

## Data Availability

The original contributions presented in this study are included in the article. Further inquiries can be directed to the corresponding author.
